# Testicular microenvironment disruption in varicocele: mechanisms and implications for spermatogenesis

**DOI:** 10.3389/fendo.2026.1751903

**Published:** 2026-04-15

**Authors:** Dongyue Ma, Anmin Wang, Hongyuan Chang, Yongqing Zhao, Wenguang Zhou, Shengjing Liu, Jun Guo, Fu Wang, Ming Zhao, Boda Guo

**Affiliations:** 1 Xiyuan Hospital of Clinical Medical College, Beijing University of Chinese Medicine, Beijing, China; 2 Department of Andrology, Xiyuan Hospital of China Academy of Chinese Medical Sciences, Beijing, China; 3 Department of Andrology, Wangjing Hospital of China Academy of Chinese Medical Sciences, Beijing, China; 4 Department of Urology, Peking University Third Hospital, Beijing, China

**Keywords:** inflammation, oxidative stress, spermatogenesis impairment, testicular dysfunction, varicocele

## Abstract

Varicocele is a common cause of male infertility, but the mechanisms by which it disrupts testicular homeostasis and impairs spermatogenesis remain incompletely elucidated. This article reviews current evidence on the multifactorial disturbances in the testicular microenvironment induced by varicocele, with a focus on hemodynamic, biochemical, and structural abnormalities. Anatomical predisposition, venous valve incompetence, and impaired venous return collectively lead to chronic venous hypertension, causing progressive dilation of the pampiniform plexus and severe hemodynamic dysregulation. These primary vascular abnormalities subsequently establish a foundation for downstream cellular damage, including testicular hyperthermia, hypoxia, and metabolic stress. Among these pathological processes, oxidative stress is widely recognized as a central mediator of testicular injury. Excessive reactive oxygen species overwhelm intrinsic antioxidant defenses, disrupt mitochondrial function, damage germ cell DNA, and impair epididymal sperm maturation, ultimately leading to reduced sperm concentration, motility, and viability. Simultaneously, elevated inflammatory cytokines and immune dysregulation further compromise Sertoli and Leydig cell function, activate inflammasome signaling and amplify inflammatory injury. These inflammatory signals also synergize with oxidative damage to disrupt the blood–testis barrier, resulting in increased permeability, autoimmune activation, and accelerated loss of germ cells. Structural impairment of the seminiferous epithelium, mitochondrial dysfunction, and the activation of intrinsic and extrinsic apoptotic pathways further exacerbate spermatogenic failure. Ultimately, varicocele induces a multifaceted and sustained cycle of testicular microenvironment disruption, impairing spermatogenesis at multiple levels—from Sertoli cell function and blood–testis barrier integrity to germ cell survival and sperm DNA stability.

## 1 Introduction

Varicocele, defined as the abnormal dilatation and tortuosity of the pampiniform venous plexus within the spermatic cord, is recognized as one of the most prevalent causes of male infertility (1, 2). It affects approximately 15% of the general male population and up to 40% of men presenting with primary infertility (3). Despite its high incidence, the precise mechanisms by which varicocele impairs testicular function and spermatogenesis remain incompletely understood (4).

Current evidence suggests that varicocele is not merely a benign vascular anomaly but rather a complex pathological condition involving multifactorial disruptions in the testicular microenvironment (5). These disruptions include thermal stress, oxidative damage, hypoxia, and impaired hormonal signaling, all of which may synergistically compromise the structural and functional integrity of the seminiferous epithelium (6, 7).

This review aims to elucidate the underlying mechanisms by which varicocele disrupts the testicular microenvironment, with particular emphasis on anatomical and hemodynamic abnormalities, altered testicular perfusion and oxygenation, and their downstream effects on spermatogenesis. Understanding these pathophysiological processes is crucial for the development of targeted diagnostic markers and effective therapeutic interventions.

## 2 Etiology of varicocele

### 2.1 Anatomical predisposition

Anatomical predisposition is considered one of the fundamental determinants in the development and progression of varicocele. The unique anatomical configuration of the left testicular vein confers a pronounced hemodynamic vulnerability, rendering it particularly susceptible to pathological changes (8). Unlike the right testicular vein, which drains directly into the inferior vena cava, the left testicular vein generally follows a longer course and enters the left renal vein at an almost perpendicular angle. Moreover, before draining into the inferior vena cava, the left renal vein traverses the space between the abdominal aorta and the superior mesenteric artery. Under certain conditions, this anatomical termination and its relationship with surrounding structures may restrict venous outflow, thereby further impeding blood return from the left testicular vein.

This mode of terminal venous drainage readily compromises the efficiency of venous return, exposing the pampiniform plexus to a chronically elevated venous pressure environment, which in turn promotes blood stasis and progressive venous dilation (9). Therefore, the anatomical susceptibility of left-sided varicocele should be understood as the combined result of multiple factors, including the venous drainage pathway, angle of venous entry, vein length, and regional venous pressure distribution. These anatomical and hemodynamic features provide the structural basis for subsequent venous wall remodeling, progressive valvular dysfunction, and the sustained progression of varicocele. Collectively, these mechanisms offer a plausible explanation for epidemiological observations consistently reporting that approximately 80%–90% of varicocele cases occur on the left side (8).

### 2.2 Incompetence of venous valves

**Figure 1:**
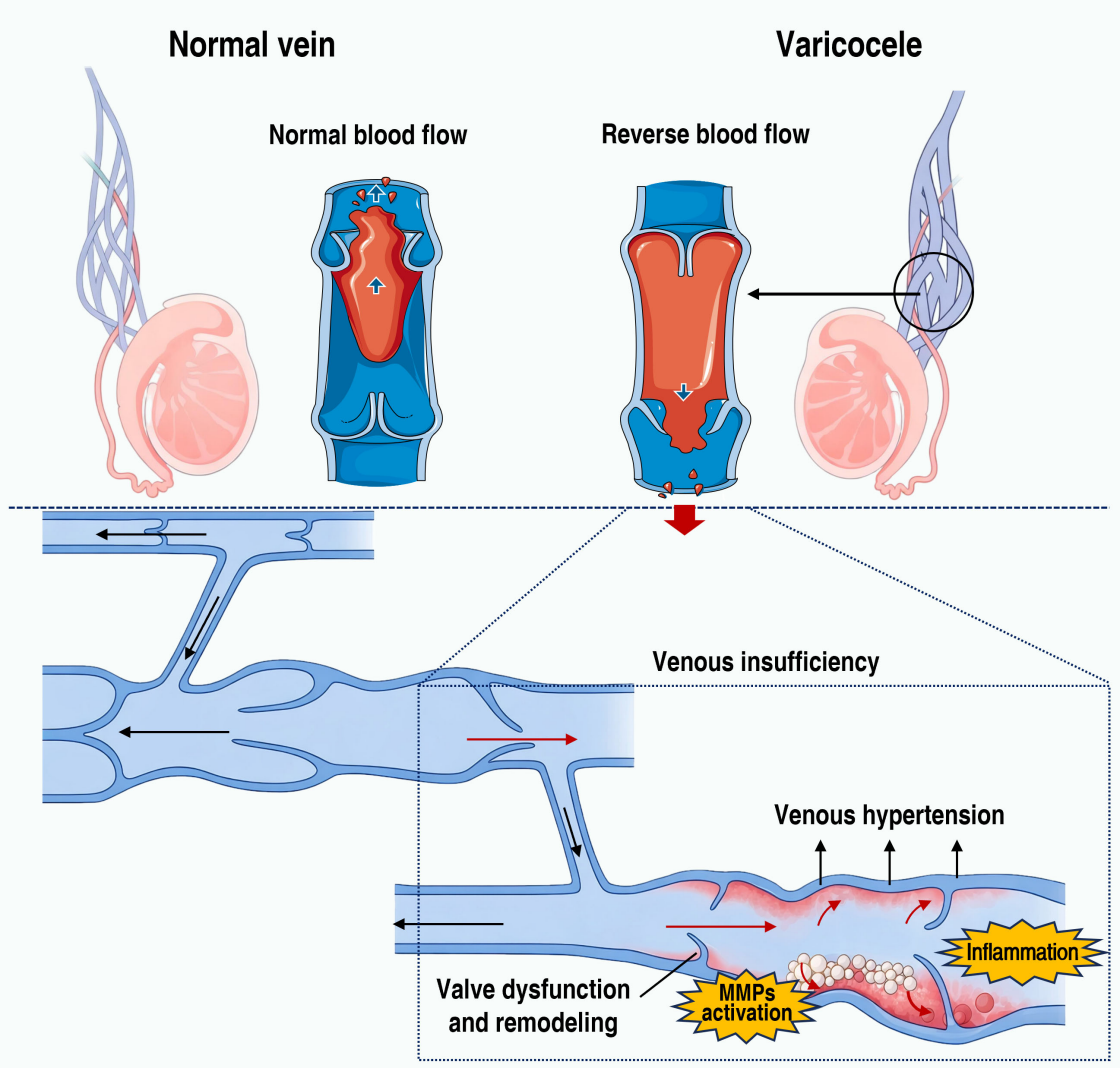
Normal spermatic vein and varicocele. MMPs, matrix metalloproteinases.

Incompetence of venous valves is recognized as a critical and direct driving factor in the development of varicocele (10). Under normal conditions, valves within the testicular venous system prevent retrograde flow during standing or episodes of increased abdominal pressure, thereby maintaining unidirectional drainage. However, many patients exhibit congenital absence of valves, reduced valve leaflets, poorly developed fibrous support, or valvular laxity, which renders the venous system incapable of resisting physiological pressure fluctuations (11). The resultant recurrent retrograde flow leads to sustained venous hypertension and progressive dilation of the pampiniform plexus (**Figure 1**).

Doppler ultrasonography commonly reveals prolonged reflux duration in the standing position, confirming the presence of a stable pathological reflux circuit driven by valvular dysfunction (12). Some studies further suggest that altered collagen content within the venous wall may contribute to insufficient structural support of valve leaflets (13). With long-term pressure overload, valvular tissue undergoes mechanical fatigue and becomes unable to achieve complete closure, allowing the reflux segment to gradually extend toward the main trunk of the testicular vein (14). Incompetent valves may be congenital but can also be exacerbated by acquired factors such as chronic elevation of intra-abdominal pressure, heavy physical exertion, or acute venous distension, all of which may cause structural deformation of valve leaflets and permanent loss of competence.

### 2.3 Impaired venous return

Impaired venous return represents the core hemodynamic foundation underlying the pathogenesis and progression of varicocele (15). This mechanism is driven primarily by upstream venous hypertension and dynamic fluctuations in intra-abdominal pressure, which collectively expose the testicular venous system to sustained high-pressure and reflux conditions (16).

Renal or caval hypertension has been identified as particularly critical in left-sided varicocele. Extensive imaging and hemodynamic evidence indicates that compression of the left renal vein between the aorta and the superior mesenteric artery (the Nutcracker phenomenon) significantly elevates renal venous pressure, which is then transmitted directly to the left testicular vein, inducing retrograde flow toward the low-resistance pampiniform plexus (17). Similarly, inferior vena cava stenosis, thrombosis, or extrinsic compression can impede bilateral testicular venous drainage, establishing chronic venous hypertension (18). Compared with the more prevalent left-sided varicocele, bilateral and isolated right-sided varicoceles occur less frequently, and their presence may suggest underlying retroperitoneal pathology, pelvic masses, or other pathological processes causing compression or obstruction of the gonadal venous outflow tract (19).

Increased intra-abdominal pressure serves as an important dynamic factor that exacerbates impaired venous return (20). Because the pelvic and abdominal venous systems communicate extensively with the testicular vein, any acute or chronic rise in abdominal pressure—such as heavy lifting, resistance training, running, or manual labor—impedes venous drainage and produces abrupt spikes in testicular venous pressure (21). Clinical observations also show that chronic cough, constipation, obesity, or bladder outlet obstruction, all of which repeatedly elevate abdominal pressure, can aggravate preexisting venous hypertension and accelerate disease progression (22, 23).

### 2.4 Genetic susceptibility

The pathogenesis of varicocele may also involve genetic factors. Studies have shown that among first-degree male relatives (brothers and fathers) of men with clinically diagnosed varicocele, the prevalence of palpable varicocele detected on physical examination reaches 33.9%, which is significantly higher than the 12% observed in control populations. This corresponds to an approximately threefold increased risk compared with controls, indicating a potential familial aggregation of the disease (24). Another study reported that the prevalence and surgical intervention rate among fathers of patients with severe varicocele (grades 2–3) were significantly higher than those of patients with mild disease, suggesting a hereditary predisposition for severe forms of varicocele (25).

Polymorphisms in glutathione S-transferase (GST) and nitric oxide synthase (NOS) genes have emerged as focal points in genetic studies of varicocele (26). Meta-analyses indicate that the overall frequencies of the glutathione S-transferase mu 1 (GSTM1) and glutathione S-transferase theta 1 (GSTT1) genotypes do not differ significantly between patients with varicocele and healthy controls; however, deletion of the GSTM1 gene is associated with exacerbated oxidative stress–related damage in affected individuals, and polymorphisms in these genes may predict postoperative outcomes following varicocelectomy (27).

In addition, specific single nucleotide polymorphisms (SNPs) in the nitric oxide synthase 3 (NOS3) gene, such as rs1799983 and rs2070744, show significantly altered distributions in patients with varicocele, suggesting that these variants may contribute to venous dilation by modulating nitric oxide (NO) bioavailability (28). Naderi et al. further proposed a potential association between varicocele and aberrant DNA and N6-methyladenosine (m6A) RNA methylation patterns, particularly global hypomethylation (2). In a clinical study of infertile men with varicocele, Arya et al. demonstrated significant DNA methylation alterations at the mitochondrial D-loop and promoter regions of mitochondria-related genes, including *UQCRC2*, *MIC60*, *TOM22*, and *LETM1* (29). Notably, these aberrant methylation patterns were partially restored following varicocelectomy or antioxidant therapy, although the degree of restoration varied across individual CpG sites (29). Taghian Dinani et al. employed a varicocele-induced rat model to dissect the mechanistic basis of global DNA hypomethylation. They observed a marked reduction in 5-methylcytosine (5-mC) signals across testicular spermatogenic cells, accompanied by a significant increase in 5-hydroxymethylcytosine (5-hmC)—an active DNA demethylation intermediate. This epigenetic shift was paralleled by a pronounced upregulation of ten-eleven translocation 2 (TET2) mRNA and protein expression, strongly implicating TET2-mediated DNA demethylation as a key driver of the hypomethylated state in varicocele (30).

Nevertheless, to date, no specific genetic determinants have been identified that can fully account for the heritability of varicocele or its clinical grading, and the precise roles of these candidate genes within sperm-related signaling pathways remain to be elucidated.

## 3 Pathological manifestations of varicocele

### 3.1 Progressive dilation of the pampiniform plexus

Progressive dilation of the pampiniform venous plexus represents the characteristic pathological hallmark of varicocele (31). Chronic venous hypertension leads to persistent venous dilatation, tortuosity, and elongation, with the venous diameter often increasing to two to three times that of normal veins (32). On physical examination, enlarged and tortuous veins can usually be palpated, whereas imaging modalities such as Doppler ultrasonography or venography typically demonstrate beaded, clustered, or reticular patterns of venous dilatation. As the disease progresses, venous enlargement becomes more pronounced in the upright position or during the Valsalva maneuver, highlighting the direct dependence of venous dilation on intraluminal pressure.

In essence, varicocele represents the manifestation of dilated venous disease within the male reproductive system, sharing core pathophysiological mechanisms with chronic venous insufficiency. Varicocele exhibits substantial similarities to lower extremity varicose veins, pelvic venous insufficiency, and hemorrhoidal disease, all of which are characterized by common underlying abnormalities of the venous wall, including venous hypertension, structural alterations of the vessel wall, venous valve incompetence, and concomitant inflammatory responses (33). Venous dilatation is largely irreversible and tends to progress with advancing age. As the central morphological feature of varicocele, progressive venous expansion provides the structural basis for subsequent hemodynamic disturbances and venous wall remodeling (34, 35). A prospective study conducted by Misra et al. (including 90 infertile men) demonstrated that the incidences of azoospermia (46.7% vs. 20.0%; OR = 3.51) and oligozoospermia (60.0% vs. 35.6%; OR = 2.71) were significantly higher in patients with varicocele than in the control group. Ultrasonographic measurements further confirmed a significant increase in the diameter of the left pampiniform plexus in the varicocele group (mean 2.95 mm). Moreover, higher-grade left-sided lesions were significantly associated with reductions in sperm concentration, motility, and other semen parameters, suggesting that the degree of venous dilatation may be positively correlated with the severity of spermatogenic impairment (36).

### 3.2 Hemodynamic abnormalities

Varicocele is closely associated with multiple forms of hemodynamic abnormalities; however, its manifestations are highly heterogeneous, and not all patients exhibit the same flow patterns (37). Clinical and Doppler ultrasonographic studies have shown that common abnormalities include prolonged venous reflux duration, reduced blood flow velocity, and variability in the timing and persistence of reflux, reflecting the combined effects of spermatic vein valvular incompetence, decreased venous compliance, and sustained venous hypertension (38). In a subset of patients, continuous and spontaneous reflux can be observed in the upright position, indicating the presence of persistent retrograde blood flow even at rest (38). When blood from the left renal vein refluxes downward through the left internal spermatic vein, blood at core body temperature is directly delivered into the scrotum, thereby attenuating or even disrupting the countercurrent heat exchange mechanism of the pampiniform plexus.

In contrast, another subset of cases is not characterized by overt reflux but instead exhibits markedly reduced blood flow velocity or even near-stagnant venous stasis (39). These distinct hemodynamic patterns may represent different stages of varicocele progression or arise from differing pathological backgrounds, and their impacts on testicular structure and function may therefore vary.

Venous stasis and/or persistent reflux can further elevate intraluminal pressure within the spermatic veins, leading to progressive vascular dilation and increased wall stress. Chronic exposure to elevated venous pressure is thought to induce structural remodeling of the venous wall, manifested as irregular luminal dilation, which in turn exacerbates local flow turbulence and markedly alters the distribution of shear stress acting on the vessel wall (40). Abnormal shear stress has been shown to impair endothelial function, reduce the bioavailability of vasoactive mediators such as nitric oxide, and weaken venous contractile and regulatory capacity (41, 42). These hemodynamic disturbances are generally progressive, potentially worsening with advancing age and prolonged disease duration, may emerge as early as adolescence, and often deteriorate further in adulthood. Overall, abnormal spermatic venous hemodynamics do not represent a single pattern but rather constitute a dynamic and heterogeneous pathological process that forms an important basis for downstream testicular structural and functional impairment in varicocele.

### 3.3 Structural changes of the venous wall

Structural remodeling of the venous wall represents a hallmark histopathological feature of chronic varicocele. Persistent venous hypertension and abnormal hemodynamics drive profound structural and cellular remodeling of the spermatic vein wall (43). Human histological studies have demonstrated that varicose spermatic veins exhibit disorganized hyperplasia of smooth muscle cells, thickened and clumped collagen fibers, and ultrastructural mitochondrial vacuolization; concurrently, osteopontin (OPN) expression in smooth muscle cells increases with vein diameter, whereas α-smooth muscle actin (α-SMA) expression remains largely unchanged, suggesting a phenotypic shift from contractile to secretory, remodeling-prone smooth muscle cells (44). *In vitro* studies support these findings, showing that smooth muscle cells derived from varicose veins display marked dedifferentiation, enhanced proliferative and migratory capacity, increased matrix metalloproteinase-2 (MMP-2) production, and elevated collagen synthesis, accompanied by downregulation of contractile phenotype markers, collectively promoting extracellular matrix turnover and venous wall remodeling, thereby weakening mechanical resistance to chronic pressure (45). Moreover, varicocele-associated hemodynamic disturbances, including elevated intraluminal pressure and altered shear stress, activate vascular endothelial cells and induce expression of adhesion molecules such as VCAM-1 and ICAM-1, initiating and sustaining chronic inflammation that contributes to progressive venous dysfunction (46).

### 3.4 Secondary structural alterations and semen impairment in varicocele

Progressive dilation of the pampiniform plexus and venous reflux in varicocele can induce structural and functional alterations in surrounding tissues. These changes are often associated with testicular microcirculatory abnormalities and reduced testicular volume, reflecting the impact of chronic venous congestion on testicular morphology and function (47). While most patients with varicocele are asymptomatic, some may experience testicular, scrotal, or inguinal discomfort, as well as a sensation of scrotal heaviness. Varicocele may also be linked to systemic vascular alterations, including impaired flow-mediated dilation in peripheral arteries and an increased prevalence of concomitant venous abnormalities in the lower limbs and pelvis (33). Furthermore, patients with varicocele exhibit a higher incidence of lower-limb venous symptoms, such as pain, burning, swelling, and pruritus, with symptom severity correlating with varicocele grade (48).

Existing evidence has demonstrated that varicocele leads to a progressive, time-dependent decline in semen parameters (49). A case-control study revealed that individuals with varicocele exhibit global sperm DNA hypomethylation, along with region-specific methylation alterations associated with spermatogenesis, meiosis, and semen quality, suggesting an epigenetic mechanism underlying impaired sperm function (50). Compared to men without varicocele, those with varicocele show significantly reduced sperm concentration, total sperm count, progressive motility, and normal morphology, alongside elevated sperm DNA fragmentation index and markers of oxidative stress (51). The severity of varicocele is positively correlated with the degree of impairment in semen parameters, indicating a progressive impact on spermatogenic function (19). Furthermore, even in patients with subclinical or low-grade varicocele, varying degrees of occult impairment in semen quality can occur, supporting the notion that varicocele exerts a detrimental effect on spermatogenesis through chronic alterations in the testicular microenvironment (52). In addition, multiple studies have reported that surgical intervention significantly improves sperm concentration, normal morphology, and motility (53, 54).

## 4 Varicocele-induced disruption of the testicular microenvironment

**Figure 2:**
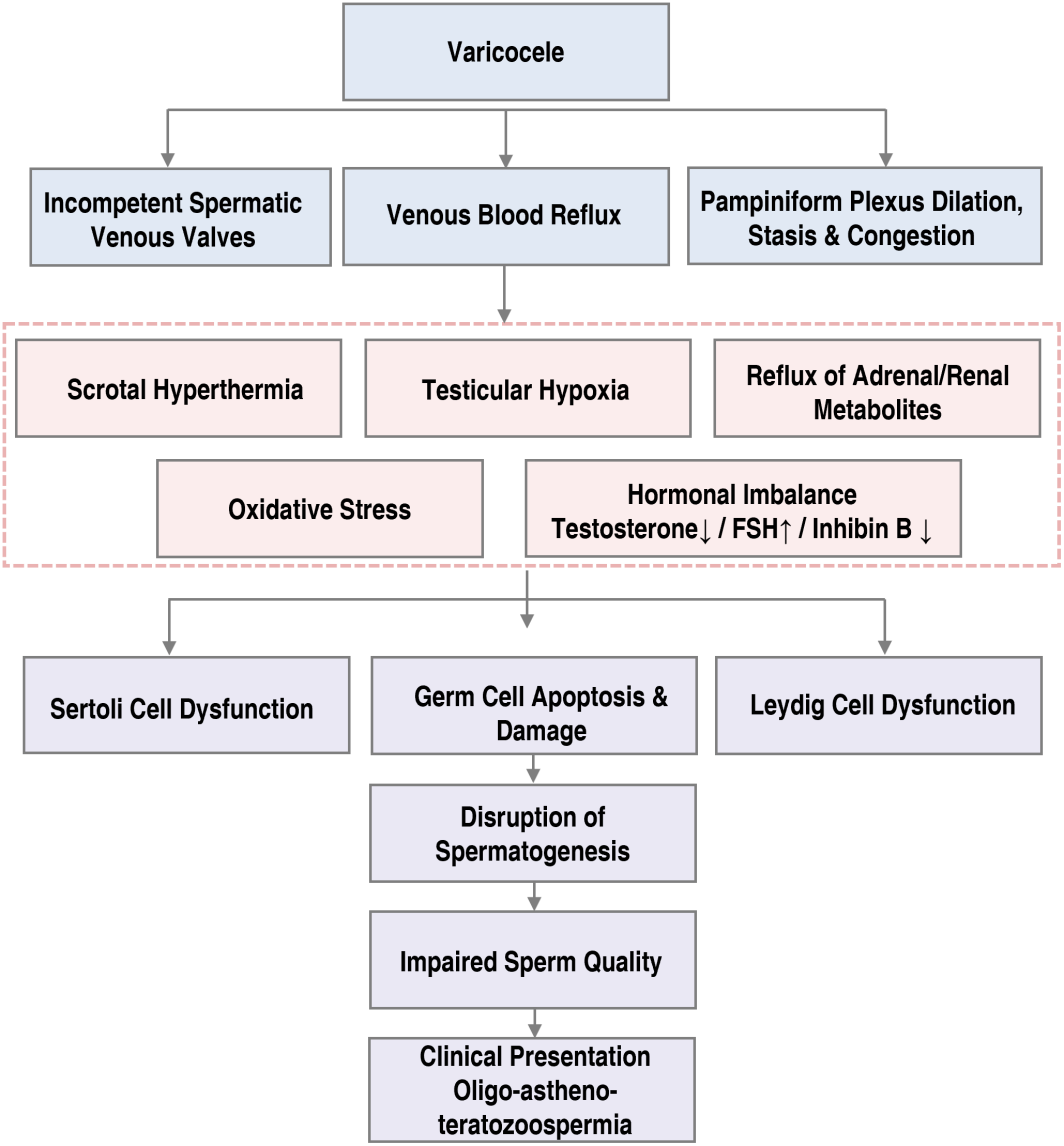
Overview of the mechanism by which varicocele affects spermatogenesis in males.

Varicocele leads to a cascade of biochemical and structural abnormalities within the testis (**Figure 2**). Hemodynamic alterations—such as venous hypertension, stasis, and retrograde reflux—promote excessive heat accumulation, hypoxia, and metabolic stress. Varicocele primarily impairs testicular function by elevating intra-testicular temperature. This occurs because the condition disrupts the countercurrent heat exchange mechanism between the pampiniform plexus and the central arterial system, resulting in reversed venous flow (55). These pathological changes trigger oxidative damage, inflammatory activation, endocrine disturbances, and structural disintegration of the seminiferous epithelium, ultimately impairing spermatogenesis.

### 4.1 Oxidative stress and ROS accumulation

Varicocele induces hemodynamic disturbances that lead to increased testicular temperature, hypoxia, and metabolic stress, thereby triggering a cascade of biochemical and structural injuries, among which oxidative stress represents the most central and decisive pathological mechanism (56). Under physiological conditions, reactive oxygen species (ROS) are maintained at low levels and participate in mitochondrial activity, sperm capacitation, and chromatin stability (57). However, under heat stress, hypoxia, and venous stasis associated with varicocele, ROS production has been shown to increase, while the antioxidant defense system—including superoxide dismutase (SOD), catalase (CAT), and glutathione peroxidase (GPx)—becomes dysregulated, thereby exposing testicular tissue to an oxidative stress–dominant microenvironment (58). Heat stress is a major driver of excessive ROS generation in patients with varicocele (26). Persistently elevated and dysregulated scrotal temperature suppresses the expression of the heat shock protein HSPA2, thereby compromising the cell’s ability to defend against thermal and oxidative damage (59). This not only inhibits antioxidant enzyme activity but also accelerates ROS production, exacerbating germ cell injury—a mechanism similar to that observed in heat-related fertility disorders such as cryptorchidism (60). Notably, animal studies have further demonstrated that varicocele-induced testicular injury exhibits pronounced spatial heterogeneity. Rat model studies show that seminiferous tubules located adjacent to varicose veins are subjected to higher levels of localized oxidative stress, accompanied by a significant downregulation of DNA repair enzyme expression and the occurrence of focal cell cycle arrest, whereas seminiferous tubules located farther from the veins exhibit relatively milder damage (61).

Excessive ROS directly damage tight junctions between Sertoli cells, thereby compromising blood–testis barrier (BTB) integrity, disrupting the organization of the seminiferous epithelium, and ultimately inducing germ cell detachment and apoptosis (62). These changes collectively result in reduced sperm concentration, motility, and viability. ROS also damage epididymal epithelial cells, impairing their secretory and maturation functions and limiting the acquisition of fertilization potential during sperm transit and storage (63). Sustained oxidative stress further induces increased expression of pro-apoptotic proteins such as BAX and reduced expression of BCL-2, thereby exacerbating programmed cell death in both epididymal and testicular germ cells (64). Moreover, because sperm membranes are enriched in polyunsaturated fatty acids (such as DHA), contain minimal cytoplasm, and lack efficient DNA repair mechanisms, spermatozoa are highly susceptible to oxidative damage. Excessive ROS trigger lipid peroxidation cascades, leading to extensive loss of membrane fatty acids, reduced membrane fluidity, and impaired membrane function, along with the generation of toxic by-products such as malondialdehyde, ultimately resulting in severe cellular dysfunction (65).

### 4.2 Elevated inflammatory cytokines and immune dysregulation

Inflammatory responses and immune dysregulation have been clearly identified as major driving factors in the pathogenesis of varicocele, representing critical pathological processes that contribute to testicular injury and sperm dysfunction following oxidative stress (66). Numerous studies have reported significantly elevated levels of pro-inflammatory cytokines in the seminal plasma and testicular tissue of men with varicocele (5, 67). These cytokines disrupt Sertoli cell tight junctions, reduce Leydig cell steroidogenic capacity, and induce germ cell apoptosis, collectively impairing the testicular microenvironment both structurally and functionally. In addition, varicocele compromises testicular immune privilege, leading to increased infiltration of macrophages and activated T lymphocytes, which amplifies local immune responses and promotes the persistence and progression of inflammatory injury (68). Animal studies further demonstrate marked activation of the NLRP3 inflammasome in varicocele models, indicating that innate immune pathways contribute to inflammatory amplification and apoptosis regulation, thereby accelerating testicular damage (69).

Leukocytospermia is another characteristic manifestation of varicocele-associated inflammation, defined by an increased number of leukocytes in seminal fluid and frequently accompanied by reduced fertility (70). Once leukocytes enter the semen, they can actively or passively generate ROS, overwhelming intrinsic antioxidant defenses and further elevating ROS levels. This exacerbates lipid peroxidation, mitochondrial DNA damage, and reduced ATP production, ultimately decreasing sperm motility. Clinical evidence shows a negative correlation between leukocyte concentration and normal sperm morphology, while *in vitro* studies confirm that cytokines such as IL-6, TNF-α, and IL-1β directly impair sperm motility and mitochondrial function (71). In patients with varicocele, increased levels of polymorphonuclear leukocytes (PMNs) in semen are significantly negatively correlated with the activity of the epididymal marker neutral α-glucosidase (NAG), while NAG activity is positively associated with sperm fertilizing capacity, suggesting that epididymal inflammation may contribute to sperm dysfunction by impairing epididymal function (72). Moreover, elevated seminal IL-1β levels are strongly associated with decreased mitochondrial activity in sperm, suggesting that pro-inflammatory cytokines not only originate from testicular inflammation but also act within the seminal microenvironment to damage mature sperm (73).

### 4.3 Blood–testis barrier disruption and increased permeability

Varicocele–induced impairment of male reproductive function extends beyond alterations in semen quality and penetrates deeper into the testicular microenvironment, in which disruption of the BTB represents a critical pathological event. The BTB is formed by peritubular myoid cells, the basement membrane, and tight junctions between Sertoli cells, and its primary function is to provide a stable, immune-privileged microenvironment for spermatogenesis. Under varicocele conditions, the structural and functional integrity of this highly specialized barrier is markedly compromised.

**Figure 3:**
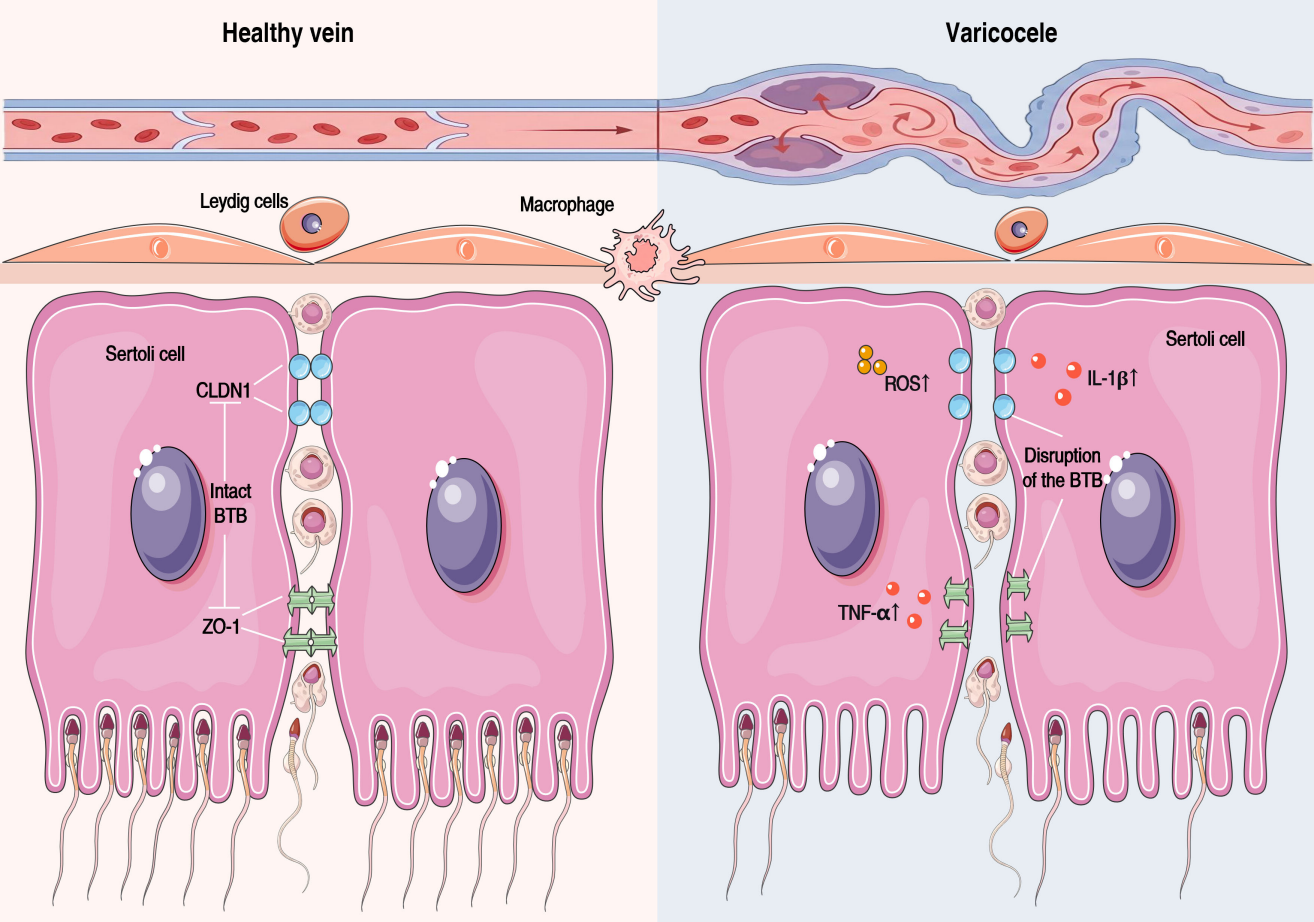
Disruption of the BTB tight junctions induced by varicocele. CLDN1, claudin-1; BTB, blood–testis barrier; ZO-1, zonula occludens-1; TNF-α, tumor necrosis factor-alpha; IL-1β, interleukin-1 beta.

Evidence from animal models demonstrates that varicocele can induce structural abnormalities of the BTB and even lead to the presence of lymphocytes sensitized to sperm antigens in lymphoid organs, indicating a breakdown of local immune tolerance (74). At the molecular level, testicular tissues from patients with varicocele exhibit significant downregulation of key proteins involved in BTB formation and maintenance, including E-cadherin, α-catenin, and Claudin-11, which are essential components of the Sertoli cell tight junction complex (75). These proteins play indispensable roles in intercellular adhesion, signal transduction, and barrier function; their reduction directly undermines the structural and functional integrity of the BTB (**Figure 3**).

The underlying mechanisms may be related to varicocele-associated pathological factors—such as venous stasis, local hyperthermia, and oxidative stress—which trigger alterations in intracellular signaling pathways. For example, varicocele-induced upregulation of protein kinase C-1 receptor activity may disrupt BTB integrity and the dynamic equilibrium of apical ectoplasmic specializations by modulating focal adhesion kinase phosphorylation (76).

Increased BTB permeability has dual pathological consequences. On the one hand, immune cells or immune mediators may more readily penetrate the barrier, directly attacking and damaging germ cells. On the other hand, sperm antigens within the seminiferous tubules may leak into the systemic circulation, thereby inducing the production of antisperm antibodies (ASAs) (5). Clinical observations have shown a higher prevalence of ASAs among infertile men. Although the direct causal relationship between ASAs and infertility remains controversial, the World Health Organization recommends ASA testing in cases presenting with sperm agglutination, and accumulating evidence suggests that ASAs may adversely affect semen parameters (56).

Collectively, BTB disruption represents a key mechanism by which varicocele induces testicular immune microenvironment imbalance and autoimmune responses, and it may also directly contribute to impaired spermatogenesis (77). Nevertheless, the extent to which BTB integrity loss directly leads to reduced spermatogenic capacity requires further elucidation.

### 4.4 Increased germ-cell apoptosis and spermatogenic failure

Spermatogenesis in mammals is a delicate balance of cell proliferation, differentiation, and apoptosis (78). The normal testis, located within the scrotum, maintains an optimal temperature and relatively independent blood oxygen supply. However, varicocele disrupts this equilibrium. Spermatogenesis is highly sensitive to temperature, and the heat stress induced by varicocele is interrelated with excessive ROS and increased apoptosis, with genetic factors also playing a role. In both human testes and animal models, upregulated expression of apoptosis markers, including Bax, caspase-3, and cytochrome c release, has been observed (79).

In this process, the upregulation of the cellular stress marker p53 can induce cell cycle arrest or apoptosis. In rat models, elevated testicular p53 expression leads to apoptosis of spermatogonia and primary spermatocytes, while in humans, its upregulation causes spermatogonial cell cycle arrest, though its precise role requires further clarification (80). Related experiments indicate that the Fas/FasL system plays a significant role in apoptosis in experimental varicocele rats, and inhibin B may reflect spermatogenic function (81).

Oxidative damage and disruption of the BTB expose germ cells to pro-apoptotic stimuli, while reduced support from Sertoli cells accelerates apoptosis. Heat stress also activates the mitochondrial apoptosis pathway (82). Together, these factors contribute to reduced sperm output, increased DNA fragmentation, and abnormal sperm morphology.

### 4.5 Endocrine imbalance of the gonads

**Figure 4:**
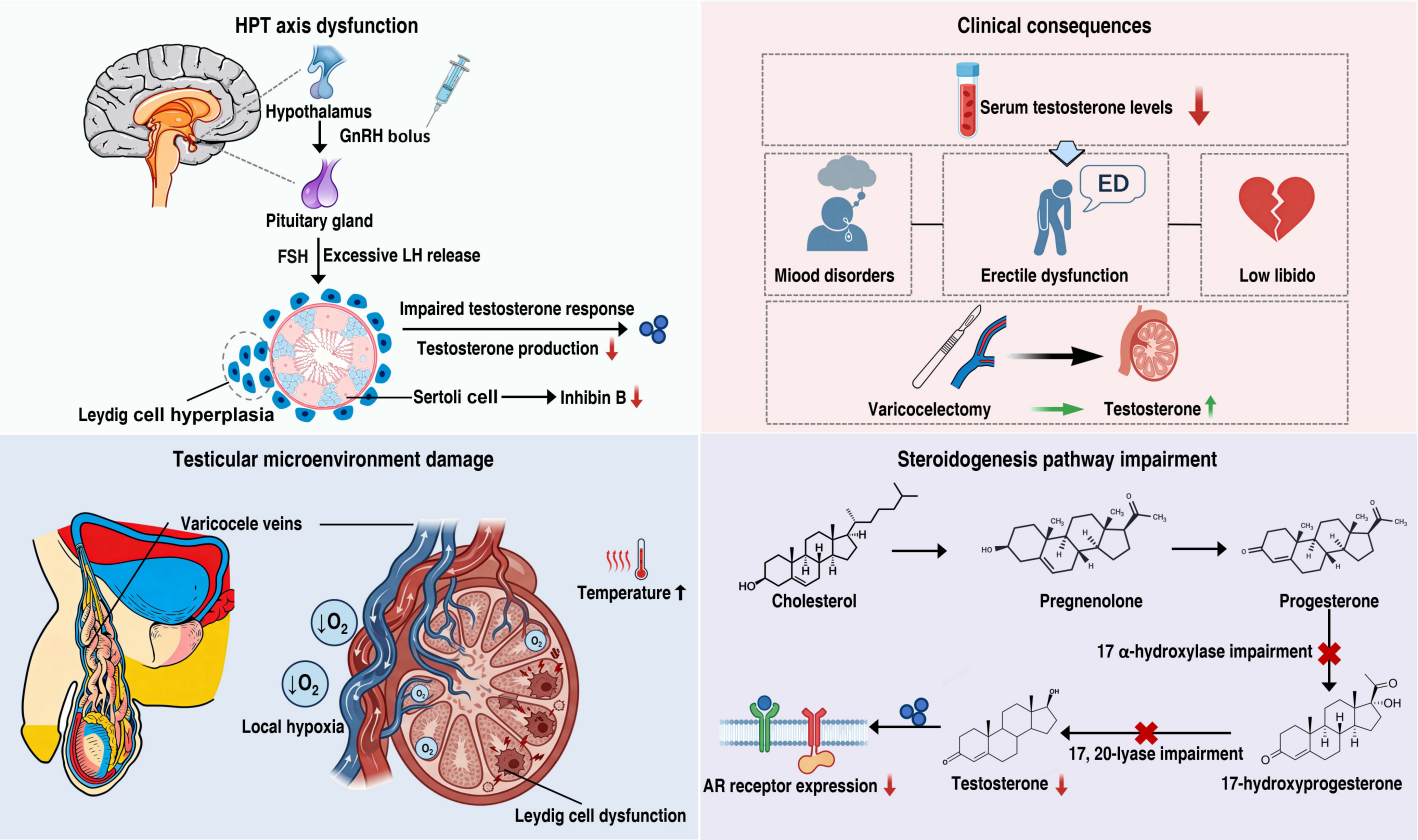
Mechanisms of endocrine imbalance in varicocele.

Varicocele can induce both systemic and intratesticular endocrine dysfunction, which constitutes one of the core pathological mechanisms underlying varicocele-associated male infertility (**Figure 4**). These disturbances are mainly manifested as dysregulation of hypothalamic–pituitary–testicular (HPT) axis feedback and impaired intratesticular testosterone synthesis. Because impaired venous drainage simultaneously leads to local hypoxia and elevated scrotal temperature, Leydig cells exposed to this unfavorable microenvironment may exhibit stress responses and functional impairment similar to those observed in Sertoli cells and germ cells.

Animal studies have demonstrated that varicocele compromises Leydig cell function by increasing apoptosis and suppressing the expression of steroidogenic acute regulatory (StAR) protein (83). Testicular biopsy specimens from patients with varicocele further confirm that, even in unilateral disease, bilateral Leydig cells may exhibit functional or morphological alterations (such as hyperplasia or atrophy), accompanied by reduced *in vitro* testosterone-producing capacity (84). Compared with healthy men, patients with varicocele generally present with lower serum testosterone levels (85, 86). Testosterone deficiency not only exacerbates spermatogenic impairment but is also associated with a spectrum of clinical manifestations, including sexual dysfunction (decreased libido and erectile dysfunction) and psychological or emotional disturbances (87, 88). Varicocelectomy provides compelling evidence for the reversibility of endocrine dysfunction, as multiple studies have shown significant postoperative increases in serum testosterone levels, particularly in patients with low preoperative levels (89, 90). In addition, clinical investigations indicate that symptoms of premature ejaculation and erectile dysfunction may improve after surgical repair of varicocele (91, 92).

Within the steroidogenic pathway, inhibition of specific key enzymes has been proposed as an important mechanism contributing to reduced testosterone synthesis; however, the available evidence remains inconsistent. Studies in rat models of varicocele have shown markedly decreased intratesticular testosterone concentrations, along with significant functional impairment of 17α-hydroxylase and C17,20-lyase, leading to the hypothesis that elevated scrotal temperature directly suppresses the activity of these enzymes (93). Consistently, patients with varicocele exhibit accumulation of 17-hydroxyprogesterone and a significantly increased 17-hydroxyprogesterone-to-testosterone ratio (94). These findings suggest dysfunction of C17,20-lyase in the testosterone biosynthetic pathway, possibly accompanied by partial impairment of 17α-hydroxylase activity. Beyond enzymatic defects, abnormalities in StAR have increasingly been recognized as a critical molecular link between varicocele and androgen deficiency. Animal studies demonstrate that varicocele markedly downregulates StAR mRNA and protein expression, thereby impairing cholesterol transport to the inner mitochondrial membrane and inhibiting the rate-limiting step of steroidogenesis (83). Nevertheless, direct evidence for altered StAR expression in human testicular tissue remains limited.

In addition to reducing androgen production, varicocele may also attenuate androgen action, a concept that has gained experimental support. In rat models of varicocele, androgen receptor (AR) transcript levels in testicular tissue remain unchanged, whereas AR protein levels are significantly reduced (76). Similar findings have been reported in humans, with infertile men with varicocele exhibiting significantly lower AR expression compared with fertile controls (95).

The relationship between clinical varicocele and disorders of hormone secretion remains incompletely understood. Early human studies suggested that varicocele may adversely affect the integrity of the HPT axis. Gonadotropin-releasing hormone (GnRH) stimulation tests have shown exaggerated luteinizing hormone (LH) responses in patients with varicocele compared with healthy controls, indicating abnormal hypothalamic–pituitary negative feedback regulation (96). Notably, in men undergoing varicocele repair, the serum testosterone response to GnRH stimulation can return to normal (97). In contrast, results from human chorionic gonadotropin (hCG) stimulation tests are inconsistent, possibly due to variations in stimulation dose and timing of hormone measurements (98, 99). These discrepancies further underscore the complexity of varicocele-associated Leydig cell dysfunction and HPT axis impairment.

## 5 Conclusions

Varicocele disrupts the testicular microenvironment through multiple interrelated mechanisms, including oxidative stress, inflammation, BTB impairment, and dysfunction of Sertoli and Leydig cells, leading to germ-cell apoptosis and impaired spermatogenesis. These multifactorial disturbances collectively reduce sperm concentration, motility, and DNA integrity, thereby impairing male fertility. Although varicocele repair has been shown to improve semen parameters and reduce oxidative damage, the precise molecular and cellular mechanisms remain incompletely understood. Future research should aim to identify early biomarkers of microenvironmental injury, clarify the signaling pathways linking venous stasis to testicular damage, explore targeted interventions such as antioxidants and BTB stabilizers, and leverage multi-omics approaches to guide personalized therapies. A deeper mechanistic understanding will facilitate more effective strategies to prevent and treat varicocele-associated infertility.
